# When to Marry

**Published:** 1893-02

**Authors:** S. H. Preston


### WHEN TO MARRY.

BY S. H. PRESTON.

Physiology proclaims the proper period to be when the parties have reached the full measure of natural growth—when the physical system has become perfected. Premature marriages are becoming notoriously common in our country. American youth are growing extraordinarily precocious, and girls and boys are rushed into fictitious maturity. As a consequence, sound health among the married, beauty and vigor among the offspring, are becoming more rare with each generation.

Physiology protests. It declares that immature parents cannot transmit to progeny the proper physical or mental power. The fruits of early marriages lamentably illustrate how the ignorance and iniquities of parents are visited upon the children.

Look upon the poor, pale, puny progeny of very young parents—see withered, fragile girls, feeble in body and imbecile in intellect anything but objects of parental pride and joy—see slender, loose-jointed, broken down boys who look as though they were brothers of their father! Every day's experience verifies the fact that the children of the early married are deficient in both body and brain, and generally die young. It has passed into a proverb that "the youngest children are the smartest."

The commonest clodhopper does not expect a good crop from unripe seed. No sensible farmer ever hoped for a paying crop from unripened seed corn. O, that parents and society would exercise as much sound sense in raising children as corn! That they would show as much science and philosophy in propagating the "human form divine" as potatoes! Every stock breeder knows that cattle which are the product of parents one or two years old are not as large and fine and kind as those of fully matured parents. And it is high time that as much common sense should be brought into requisition in the raising of children as of cattle.

The whole history of human genius and greatness is in favor of late marriages, as see the few following facts:

Pitt, Fox, and Burke, were each the youngest child of their respective families. Benjamin West was the tenth child of his parents. Daniel Webster was the youngest by a second marriage, as was also Lord Bacon. Franklin was the fifteenth child of his father and the eighth of his mother; and further, he was the youngest child for five successive generations on the side of his mother. Dante was born of his father's second wife, Bella. Lorenzo de Medici, "the magnificent," was a second son. Mirandola was a younger son. Luigo Pulci was the youngest of three brothers. Tasso was the third child. His elder brother died young. Oliver Goldsmith was the youngest of five children. Coleridge was the youngest of a clergyman's numerous family. Schiller was a younger child. Robert Fulton was the third child. Richelieu was the youngest of three sons. Lord Eldon was the eighth child by a second marriage. Mirabeau was the fifth child. Oliver Cromwell was a younger son. Queen Elizabeth was born when her father, Henry VIII., was forty-two years old.

Nearly all the sovereigns of England distinguished for great abilities were younger children, or born of mature parents. Leibnitz was an only son of his father by a second wife. Litchtenberg, the great mathematician, was the eighteenth child. Charles Lamb was the youngest child, having a brother twelve years and a sister ten years older than himself. Stilling was the youngest of ten children. Madame Roland was a second child. William Wirt was the youngest of six children. Oberlin was the youngest of nine children. Richard Watson was born when his father was sixty years of age And so on.

These instances are sufficient to show that robust children and those gifted with genius generally come from parents organically matured and with constitutions consolidated.

